# Impact of Maturation and Vitrification Time of Human GV Oocytes on the Metaphase Plate Configuration

**DOI:** 10.3390/ijms22031125

**Published:** 2021-01-23

**Authors:** Irene Peinado, Isabel Moya, Paula Sáez-Espinosa, Macarena Barrera, Laura García-Valverde, Raquel Francés, Patricia Torres, María José Gómez-Torres

**Affiliations:** 1 Assisted Human Reproduction Unit, La Fe University and Polytechnic Hospital, 46026 Valencia, Spain; peinado_ire@gva.es (I.P.); imoyamarin@gmail.com (I.M.); macarenabarreragallardo@gmail.com (M.B.); lmgv25@gmail.com (L.G.-V.); raquelfrances1@gmail.com (R.F.); patriblanc81@hotmail.com (P.T.); 2 Biotechnology Department, Alicante University, 03690 Alicante, Spain; paula.saez@ua.es; 3 Energy and Memory, Brain Plasticity Unit, CNRS, ESPCI Paris, PSL Research University, 75005 Paris, France; 4 Cátedra Human Fertility, Universidad de Alicante, 03690 Alicante, Spain

**Keywords:** in vitro maturation, cryopreservation, spindle configuration

## Abstract

The combination of in vitro maturation (IVM) techniques and oocyte vitrification (OV) could increase the number of useful oocytes in different types of patients. IVM and subsequent OV is the most widely used clinical strategy. Would the results improve if we reverse the order of the techniques? Here, we evaluated survival, in vitro maturation, time to extrude the first polar body (PB), and the metaphase plate configuration of human prophase I (GV) oocytes before or after their vitrification. Specific, 195 GV oocytes from 104 patients subjected to controlled ovarian stimulation cycles were included. We stablished three experimental groups: GV oocytes vitrified and IVM (Group GV-Vit), GV oocytes IVM and vitrified at MII stage (Group MII-Vit), and GV oocytes IVM (Group not-Vit). All of them were in vitro matured for a maximum of 48 h and fixed to study the metaphase plate by confocal microscopy. According to our results, the vitrification of immature oocytes and their subsequent maturation presented similar survival, maturation, and metaphase plate conformation rates, but a significantly higher percentage of normal spindle than the standard strategy. Additionally, the extension of IVM time to 48 h did not seem to negatively affect the oocyte metaphase plate configuration.

## 1. Introduction

Oocytes vitrification (OV) protocols developed during the last decade have allowed the introduction of this technique in all assisted reproduction (AR) laboratories. This is based on the quick freezing of the oocytes in a medium with high cryoprotectants (CRP) concentrations, preventing ice crystals formation [1]. Previous studies have focused on avoiding the toxic effects of the CRP, reducing the exposure and freezing time [2,3,4]. OV has required a lengthier optimization and implantation period than embryo vitrification (EV). One of the greatest challenges of OV is the disruption of the meiotic spindle [5,6,7,8,9]. The spindle, which is formed mainly by microtubules, is responsible of chromosome segregation [10]. The microtubules are very sensitive to temperature so, therefore, oocyte preservation is associated with an increase in embryonic aneuploidies [11,12,13]. However, it is also known that this structure is dynamic and can repolymerize when the physiological conditions are recovered [14,15,16,17,18,19].

Oocyte preservation can be performed in immature oocytes or after their in vitro maturation (IVM). Conventionally, prophase I (GV) oocytes have been considered more suitable for preservation than metaphase II (MII) oocytes, since during meiosis arresting at GV, chromatin is protected in the germinal vesicle and the spindle is not formed yet [20,21,22]. Nevertheless, even if this problem was hypothetically avoided with GV oocytes, the possible alteration in the cortical granules distribution, cytoplasmic organelles, RNA, and proteins may compromise their maturation [8,23,24,25].

Immature oocytes collection and in vitro maturation are techniques with multiple potential indications: Patients at risk of ovarian hyperstimulation (OHSS) or with Polycystic Ovary Syndrome (PCOS); women that underwent invasive surgery; patients at risk of exposure to estrogens; oocyte donation program; optimization of stimulated cycles; classic in vitro fertilization (IVF) or assisted by intracytoplasmic sperm microinjection (ICSI), and fertility preservation for social reasons or cancer patients [26,27,28]. In this last group, patients for oocyte vitrification and ovarian cortex preservation are included. However, this technique is still under development, and is not a clear alternative to other AR techniques. IVM involves nuclear and cytoplasmic changes that, as previously mentioned, are necessary for the subsequent proper embryonic development. The understanding of the molecular basis of the chromosome segregation process during IVM would reveal the full potential of this technique. Consequently, the quality of the collected MII oocytes will depend not only on the intrinsic characteristics of the original GV oocyte, but also on the vitrification protocol chosen and the conditions of the IVM process. Both processes can contribute to a deficient embryonic development or to the start of apoptotic processes [29]. 

**Figure 1:**
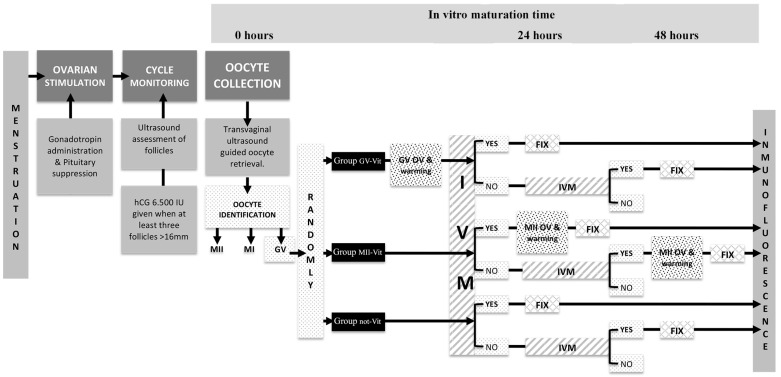
Experimental design that describes in detail the different study groups and the methodology used at the different in vitro maturation times (24 and 48 h). GV, prophase I; MI, metaphase I; MII, metaphase II, OV, oocytes vitrification; IVM, in vitro maturation; FIX, fixation; Vit, vitrification.

In the recent years, many children were born after OV [30,31,32,33], IVM [34,35,36,37,38,39], or a combination of both [40,41]. However, this combination remains controversial [42,43]. In previous studies performed by our group, the vitrification before or after the IVM for 48 h of GV did not show significant differences in some constituents of their ultrastructure [25]. In the present work, we aimed to evaluate the impact of IVM time and/or vitrification on the following rates: Survival, maturation, spindle configuration, and chromosomes distribution. To that end, we distributed the oocytes in three experimental groups: Group GV-Vit (frozen GV oocytes and then matured in vitro), Group MII-Vit (frozen MII oocytes after being matured in vitro), and Group not-Vit (GV oocytes matured in vitro and not vitrified) (see Figure 1).

Our results showed comparable vitrification survival, maturation, and metaphase plate configuration rates whether oocytes were cryopreserved before or after IVM, suggesting a possible benefit if oocytes were vitrified at GV. Furthermore, we showed that extending IVM could increase the number of available oocytes without altering their quality, at least regarding the metaphase plate configuration. 

## 2. Results

### 2.1. Survival Rate (SR)

Depending on the maturation stage on which the oocytes were vitrified, the survival rate (SR) after their warming did not show significant differences in GV (Group GV-Vit) and MII (Group MII-Vit) [Group GV-Vit 77.6% (45/58) vs. Group MII-Vit 68.2% (30/44)] (Table 1). 

Similarly, the SR was calculated according to the maturation time (24 or 48 h). For this analysis, only IVM oocytes from Group MII-Vit (*n* = 44) were selected, since Group GV-Vit maturation could be conditioned by the previous vitrification of the oocytes. This study did not show differences depending on the maturation time [24 h 65% (26/40) vs. 48 h 100% (4/4)]. 

### 2.2. Maturation Rate (MR)

Regardless of the study groups or prior state of the oocyte, fresh (Group MII-Vit + Group not-Vit) or warmed (Group GV-Vit), no significant differences were observed in maturation rates (Table 1). However, the germinal vesicle breakdown (GVBD) was significantly higher in the Group GV-Vit vs. Group MII-Vit [88.9% (40/45) vs. 71.8% (61/85); *p*-value < 0.05] (see Table 1).

The percentage of oocytes that matured during the first 24 h of culture was 62.6% (114/182), with an increase of 11% (20/182) during 25–48 h. Thus, 73.6% (134/182) of the initial GV oocytes matured after 48 h. 

### 2.3. Spindle Characteristics and Chromosome Arrangement

**Figure 2:**
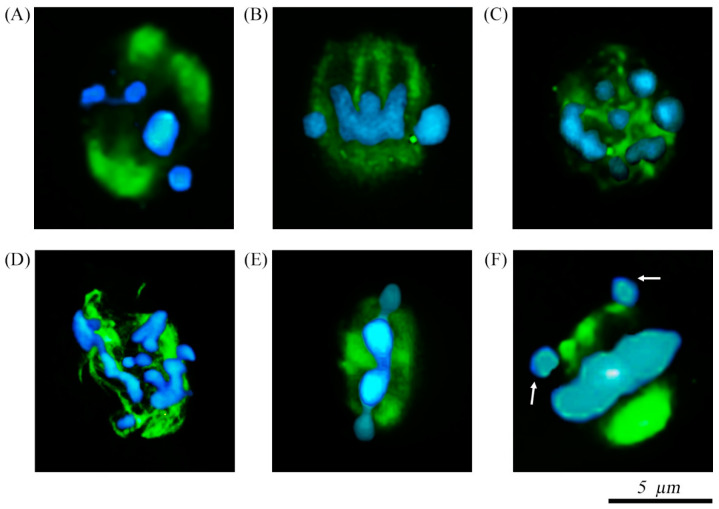
Confocal micrographs of spindle chromosome complexes. Anti-α/β-tubulin antibody and Alexa Fluor 488 to visualize the spindle (green) and DAPI to stain the chromosomes (blue). (**A**,**B**) Normal barrel-shaped spindle configuration with chromosomes arranged at the equator of the structure. (**C**) Normal spindle complex oriented perpendicular to the focal plane and chromosomes arranged in a circular way. (**D**) Spindle showing completely disorganized appearance with abnormal chromosomes configuration. (**E**) Spindle complex displaying reduction in the longitudinal dimension of the spindle and compact metaphase plate. (**F**) Normal spindle conformation with some chromosomes slightly displaced from the plane of the metaphase plate (arrows).

The reconstruction of Z-stacks sections allowed us to observe spindle configuration and chromosome alignment in each oocyte. Regarding the normal spindle conformation, if the spindle was oriented parallel to the focal plane, the complex looked as a typical barrel-shaped (Figure 2A,B). On the contrary, if the spindle was oriented perpendicular to the focal plane the fibers became circular in shape (Figure 2C). Abnormal spindles had a disorganized appearance (Figure 2D) or were shorter than usual (Figure 2E). Normal chromosomes arrangement showed the genetic material aligned in a compact metaphase plate at the spindle equator (Figure 2A,B,E) or in a perfect circle depending on the focal plane (Figure 2C). If some chromosomes were found to be slightly displaced from the metaphase plate, this configuration was classified as partially abnormal (Figure 2F) and, in the case of being completely disorganized, as abnormal (Figure 2D).

**Figure 3:**
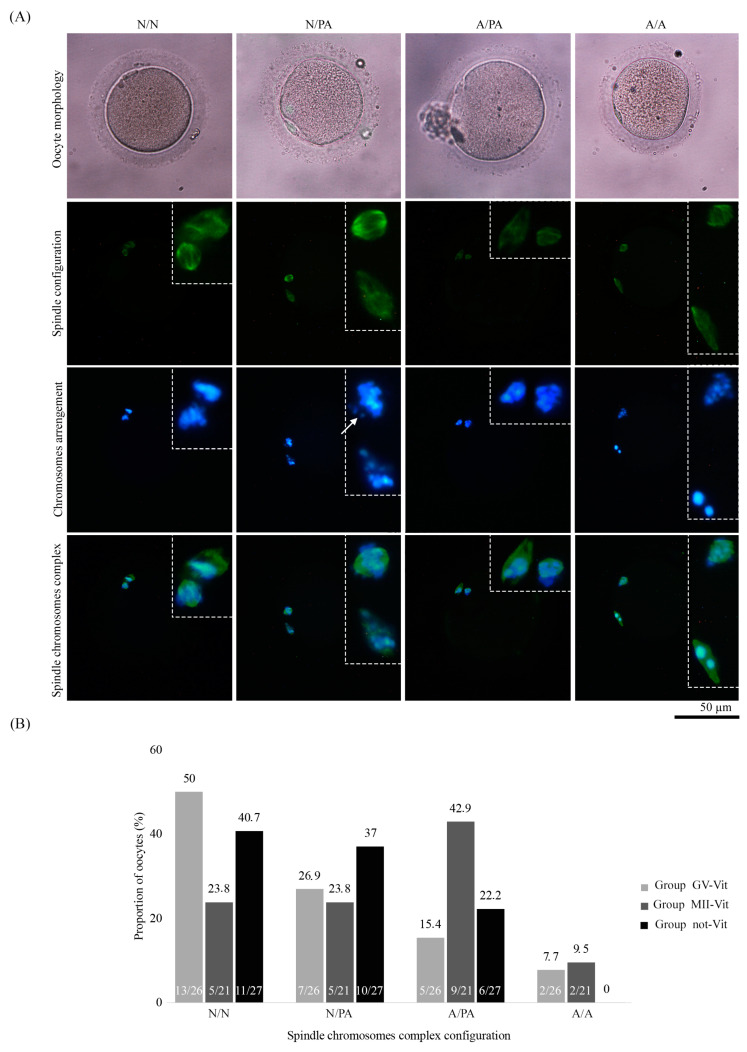
Oocyte categories according to the whole spindle chromosomes complex configuration. N/N: Polymerized spindle and normal chromosome distribution; N/PA: Polymerized spindle and partially abnormal chromosome distribution; A/PA: Depolymerized spindle and partially abnormal chromosome distribution; A/A: Depolymerized spindle and abnormal chromosome distribution. (**A**) Oocyte morphology by bright field microscopy and confocal micrographs of spindle chromosomes complexes. Anti-α/β-tubulin antibody and Alexa Fluor 488 to visualize the spindle configuration (green) and DAPI to stain the chromosomes arrangement (blue). Chromosomes slightly displaced from the plane of the metaphase plate (write arrow). In the dotted box, the mitotic spindle and chromosomes were amplified for easy viewing. (**B**) Proportion of oocytes according to their Spindle chromosomes complex configuration in the three groups of study (Group GV-Vit, Group MII-Vit, and Group not-Vit), *p*-value > 0.05. This graph also contains, inside the bar, information on the number of oocytes included in each group.

As a result of these observations, four categories regarding spindle and chromosome status were established. The first category involved oocytes with normal configuration of both structures (N/N, Figure 3A). More in detail, a barrel-polymerized spindle positioned perpendicular to the first PB and aligned chromosomes at the equator of the meiotic spindle. The second included oocytes containing a normal spindle and partially abnormal chromosomal arrangement (N/PA, Figure 3A). In this case, some chromosomes are located outside of the spindle equatorial plane (Figure 3). The third category included abnormal spindle configuration (depolymerized spindle) and partially abnormal chromosome distribution (A/PA, Figure 3A). The last category corresponded to oocytes with abnormal spindle and chromosome alignment (A/A, Figure 3A).

The comparison of the mean percentage of oocytes with normal metaphase plate configuration rate (N/N) between the three study groups did not show significant differences (see Figure 3B). No differences were found once the rest of the categories were compared (N/PA; A/PA or A/A) in the three groups. It is worth mentioning that despite not reaching statistical significance, oocytes frozen at GV (Group GV-Vit) or non-frozen (Group not-Vit) seemed to show a normal metaphase plate configuration in a higher percentage than the one obtained when the oocytes were matured and subsequently vitrified (Group MII-Vit).

**Figure 4:**
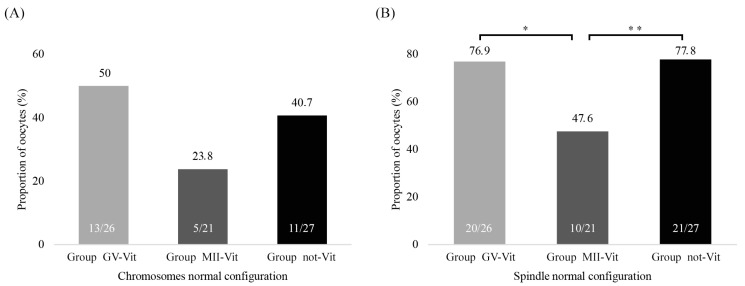
Chromosomes and spindle normal configuration of the oocytes in the three groups of study (Group GV-Vit, Group MII-Vit, and Group not-Vit). (**A**) Chromosomes normal configuration, *p*-value > 0.05. (**B**) Spindle normal configuration * *p*-value Group GV-Vit vs. Group MII-Vit = 0.038 and ** *p*-value Group MII-Vit vs. Group not-Vit = 0.03.

The study of the chromosomal distribution, regardless of the spindle conformation, did not show significant differences in any of the cases (Figure 4A). However, the exclusive evaluation of the normal spindle polymerization (barrel structure of the metaphase plate) showed significant differences between the groups studied (see Figure 4B). This significance was higher when we compared them one by one (Group GV-Vit vs. Group MII-Vit *p*-value = 0.038 and Group MII-Vit vs. Group not-Vit *p*-value = 0.030). Thus, the results exposed that MII_IVM_ oocytes that were vitrified at GV (Group GV-Vit) were similar to than those MII_IVM_ that were not vitrified (Group not-Vit), presenting a better polymerized spindle than the MII_IVM_ vitrified at MII (Group MII-Vit). 

### 2.4. Influence of In Vitro Maturation (IVM) Time on the Configuration Metaphase Plate 

Counting the 74 oocytes from the immunofluorescence study (Group GV-Vit = 26, Group MII-Vit = 27, and Group not-Vit = 21), regardless of the group that they belonged to and depending on the IVM time, 24 or 48 h, the analysis of the four different metaphase plate configurations showed significant differences between both study times [N/N: IVM24 h 39.3% (24/61) vs. IVM48 h: 38.5% (5/13); N/PA: IVM24 h 24.6% (15/61) vs. IVM48 h: 53.8% (7/13); A/PA IVM24 h 31.1% (19/61) vs. IVM48 h: 0% (0/13); A/A IVM24 h 4.9% (3/61) vs. IVM48 h: 7.7% (1/13); *p*-value < 0.05]. 

When the results were analyzed separately, the study of the chromosomal distribution, regardless of the spindle conformation, did not show significant differences between the groups (see Table 2). On the other hand, the evaluation of the spindle polymerization, regardless of the chromosomal distribution, showed a significantly higher percentage of oocytes with an optimal spindle configuration in the oocytes that matured at 48 h compared to those that did it during the first 24 h of culture (Table 2). However, these results have to be interpreted very carefully since the number of oocytes that maturated after 48 h was fairly low (*n* = 13). 

## 3. Discussion

The use of in vitro matured oocytes in reproductive cycles with or without stimulation, combined with the vitrification technique, involves novel ways of action and allows us to maximize the efficiency of the AR laboratories’ techniques. Their clinical use is conditioned to the collection of competent MII oocytes, which are able to sustain early embryonic development. Currently, the use of immature oocytes (rescue oocytes) is one of the research fields with the greatest potential to develop and optimize emerging AR techniques in humans. Therefore, there are several aspects to improve, specially IVM technique. However, there is high controversy in the literature regarding vitrification before or after IVM [42,44,45]. For that, this study was divided into different experimental groups depending on their maturation state prior to vitrification: Group GV-Vit (GV oocytes frozen and then matured in vitro), Group MII-Vit (frozen MII oocytes after being matured in vitro), and Group not-Vit (GV oocytes matured in vitro and not vitrified).

The surface/volume ratio is similar in immature (GV) oocytes or after their in vitro maturation (MII). However, the microstructure confers a priori advantages to GV oocytes over MII: (1) Chromatin protected by the nuclear membrane inside the GV and (2) absence of the microtubule assembly complex during meiosis. Nevertheless, there is no consensus in the literature about this topic [21,46,47,48,49]. Our results did not show statistical significance when comparing the survival rate of GV oocytes vs. MII_IVM_, which agreed with other studies published in humans, where survival after GV warming was comparable to the one showed by MII_IVM_, either with oocytes from stimulated [44,49,50,51] or non-stimulated [21] cycles. Therefore, in stimulated or non-stimulated cycles, we assumed that the preservation stage did not affect oocyte survival. However, it is necessary to inquiry whether their competence might be compromised. 

In this study, progression to MII at 48 h did not show significant differences depending on the previous state of the oocytes (fresh or warmed). Most of the published studies support this result, obtaining similar [52,53] or higher [44,54,55] maturation rates, if oocytes were cryopreserved at MII stage. However, a recent meta-analysis questions the fact that vitrified GV oocytes show worse results [42]. In fact, our results suggested a higher GVBD in those cases where oocytes were vitrified prior to their maturation. This agrees with previous studies, which showed that the reagents used for vitrification induce cytoplasmic changes that favor GVBD. Specifically, they indicate an increase in the intracellular levels of Ca^2+^ and intra-oocyte cAMP degradation, favoring the activation of the maturation promoting factor (MPF) and meiosis resumption [54,56]. Following this line, other research has shown that the short-term presence of the cryoprotectants dimethyl sulfoxide (DMSO) and ethylene glycol (EG) facilitate Ca^2+^ traffic, having an impact on the increase of the MR [57,58]. DMSO would mobilize the intra-oocyte Ca^2+^ deposits, especially the endoplasmic reticulum, while EG would favor the entry of extracellular Ca^2+^ inside the oocyte [58]. 

In this work, we stablished a time for IVM of 48 h, generally longer than the one recommended by most of the published literature, which is usually stablished at a maximum of 24/32 h [59,60]. Under these conditions, maturation of both vitrified and fresh GV oocytes was similar at 24 h and 48 h. This result contradicts the hypothesis that vitrified GV oocytes before their IVM need more time to extrude the first polar body [44,49]. Furthermore, in opposition to this hypothesis, the results published by Lowther and colleagues showed differences in time regarding the GVBD, but only during the first 2 h of culture, which is the time of adaptation to the physiological culture conditions re-established after the warming. However, 6.5 h later, the rates were similar, even slightly higher than the group with frozen oocytes [53]. Meanwhile, after studying if culture time affects survival rate, our results did not show significant differences when comparing Group MII-Vit MII_IVM_ oocytes at 24 vs. 48 h. Hence, contrary to what is indicated by the studies mentioned above, our results suggest that extending the culture time does not alter the structures more sensitive to cryogenic damage. 

Once the maturation obstacle is overcome and in order to continue evaluating the competence of the collected oocytes, it is necessary to evaluate the possible deficiencies that vitrification and IVM may have caused in the oocytes, and observe whether altering the order of these leads to better results. Understanding how human oocytes perform chromosomal segregation is greatly important, since errors during this process explain most of the human aneuploidies [61]. Microtubular depolymerization caused by temperature changes has been described both in mature and immature oocytes [52,62,63,64,65]. However, repolymerization of the spindle once physiological conditions are recovered has also been reported [5,66,67]. Despite this, previous studies describe that frozen oocytes, both GV and MII_IVM_, show abnormal spindles and an altered chromosomal distribution when compared to non-frozen oocytes [44,52,63]. Therefore, temporary depolymerization would be recoverable, but its functionality could be affected, resulting in an abnormal chromosomal segregation, maturation arrest, and aneuploidy formation [5].

The Configuration rate metaphase plate obtained in this study did not show significant differences between none of the groups of study when the chromosomal and spindle configuration were evaluated together. When we evaluated the spindle configuration, regardless of the chromosomal distribution, we observed significant differences in favor of non-vitrified MII_IVM_ or vitrified at GV stage and then matured, compared to oocytes that were vitrified after maturation. The time required to recover physiological conditions after warming in this study was 2 h, which is recommended by different authors [68,69,70], although others recommend a slightly higher interval [7,47,52]. Likely, the low spindle configuration rates in Group MII-Vit were due to an insufficient recovery time after warming. These results support that, after warming, the spindle could be reassembled, but in an abnormal way. Moreover, it confirms that GV vitrification does not prevent the correct formation of the spindle after their vitrification. 

In addition to cryopreservation, suboptimal culture conditions during IVM could increase alterations in the meiotic apparatus [62,71]. However, an exhaustive observation of this between MII oocytes matured in vivo and in vitro did not show variations in the actin intensity pattern in the proximal and distal domains of the metaphase spindle, but showed an increase in the cytoplasmic actin in MII_IVM_ oocytes [72]. It is possible that this reflects an adaptative response to IVM conditions to increase the cytoplasmic actin fluxes, previously described in rodent oocytes [73]. Our study did not include MII oocytes matured in vivo as a second control group for IVM because human MII oocytes matured in vivo are highly valuable in the clinic and are used exclusively for the patient. However, there are other studies that point to a worse spindle configuration and chromosome distribution in oocytes matured in vitro vs. in vivo [74,75,76]. 

Depending on the maturation time, Escrich and colleagues concluded to not exceed the culture more than 24 h, since oocytes that matured between the first 18.4 ± 2.7 h showed better activation and division rates and a lower rate of meiotic errors in comparison with the ones that did it later [59]. Nevertheless, in this work, regarding the metaphase plate configuration, we observed significant differences in favor to the oocytes that matured between 25–48 vs. 0–24 h. Therefore, according to our results, the time of culture of oocytes up to 48 h did not imply higher meiotic error rates. These would suggest, again, that extending the time of culture would be beneficial, favoring the synchronization between nuclear and cytoplasmic maturation, allowing better oocyte meiotic development. 

As already mentioned, there are contradictory results in the literature, mainly because it is a procedure that combines several techniques, each one of them with variable results depending on the conditions in which they have been performed [7,67,77,78,79,80]. In addition, oocytes generated in vivo may also present low competence due to the characteristics of the donor or the patient [81,82]. Therefore, at this point it is worthwhile to highlight that most of the published studies randomly assign the oocytes to the groups of study, but do not describe the characteristics of the women that participate in the study or the homogeneity of the groups. In the literature, we found some studies that relate vitrification, IVM, or the metaphase plate configuration with some of the clinical characteristics of the patients. For instance, they point that in vitro maturation rate is not affected by the age [83], except prepubescent or elderly women who show lower maturation rates [84,85]. However, survival to vitrification is diminished by age increase [7,81]. The increase of the age also influences on the correct organization of the metaphase plate in human oocytes. It has been reported a higher percentage of chromosome misalignment in those cases [86], due to a lack of adhesion [87] caused by a reduction of the assembly points between kinetochores and microtubules [88,89]. There is also evidence in the literature regarding the diagnosis of infertility [32,37]; for example, in women with endometriosis, a lower IVM rate, higher zona pellucida hardening, premature exocytosis of the GC, and a high disruption of the chromosomes in the metaphase plate have been described [90]. All these factors should be taken into account when conducting further studies in humans. 

Overall, vitrification and IVM techniques can alter essential structures for the proper development of the embryo. These alterations could impede meiosis, fertilization, or early embryonic development, preventing the activation of the embryonic genome and therefore, avoiding the formation of a blastocyst with implantation capacity [91,92]. Further studies in humans that allow us to define the moment and the conditions in which to perform these techniques are required to consolidate their combination and increase their range of indications. It should not be forgotten that rescue IVM may be the only gestation opportunity, without resorting to egg donation, for certain types of patients as a oncological or low response, among others. 

## 4. Materials and Methods 

This study was approved by the Institutional Review Board of the Hospital Universitario y Politécnico La Fe, Valencia, Spain. All 104 patients included in the study were fully informed and gave their signed consent to donate the 195 GV oocytes collected from their ICSI cycles carried out in the Human Reproductive Unit of the previously mentioned Hospital. 

### 4.1. Oocyte Collection

The patients were subjected to controlled ovarian stimulation following a short antagonist protocol. Pituitary suppression was performed by administration of (150–300 IU/day) rec-FSH (Gonal F 1050; Merck and Co, Madrid, Spain) and GnRH (Orgalutran^®^; MSD and Co., Hoddesdon, UK). When the growth of at least three follicles >16 mm was observed, ovulation was induced by the administration of 250 μg of rec-hCG (Ovitrelle, Merck, London, UK). Oocyte retrieval was performed 36 h after hCG administration via ultrasound-guided transvaginal puncture-aspiration. Cumulus-corona-oocyte (CCO) complexes denudation was carried out using hyaluronidase SynVitro^®^ Hyadase; Origio^®^ Solution, Màlov, Denmark) for a maximum of 30 sec with a denudation pipette (Denudation pipette Flexipet^®^, Cook^®^ Medical, Bloomington, IN, USA). Removal of cumulus-corona cells is required to evaluate and classify the oocyte nuclear maturation state. Despite coming from stimulated cycles, a total of 199 GV oocytes showed an intracytoplasmic nucleus called the germinal vesicle, characteristic of the prophase of the first meiotic division. After the IVM rescue, we included in this study all the oocytes that showed an approximately circular shape and a variable size between 120–140 μm; homogeneous or slightly heterogeneous cytoplasm with no granularity such as inclusions or refractile bodies. Four oocytes (2%) were excluded from the study for being giant, presenting dimorphisms in their zona pellucida, or showing large vacuoles or signs of atresia/degeneration in their ooplasm.

### 4.2. Experimental Design

Prospective, randomized cohort study.

To evaluate the effects of cryopreservation on oocytes in vitro maturation, the study was divided in different experimental groups depending on their maturation state prior to vitrification: Group GV-Vit (GV oocytes vitrified and then matured in vitro), Group MII-Vit (vitrified MII oocytes after being matured in vitro), and Group not-Vit (GV oocytes matured in vitro and not vitrified) (Figure 1).

### 4.3. In Vitro Maturation

**Figure 5:**
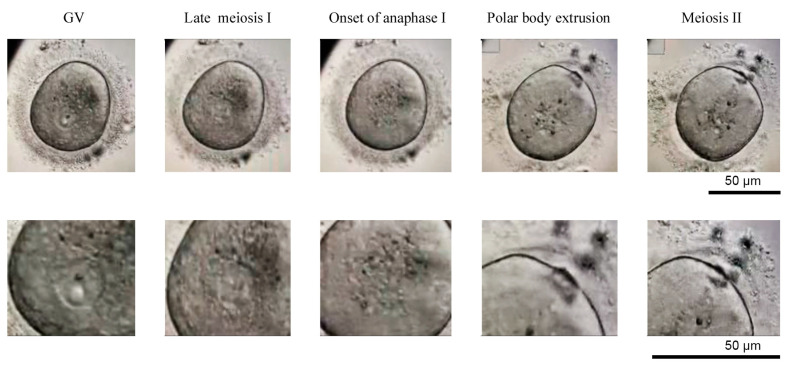
Sequence at different stages of meiosis of the human oocyte. Oocyte with an intact nucleus (prophase I, GV), late metaphase of meiosis I, onset of anaphase I, onset of polar body extrusion and meiosis II, total polar body extrusion. Images were obtained in Primo Vision Time-Lapse System (Vitrolife^®^ Göteborg, Sweden) in the Human Reproduction Unit of Hospital Universitario y Politécnico, La Fe.

GV oocytes were individually cultured in micro-drops of 25 μL of culture medium covered by mineral oil OVOIL^TM^ on 1008 plates at 37 °C and 5% CO_2_. We used SAGE 1-Step^TM^ (Origio^®^, Màlov, Denmark) culture medium, supplemented with human menopausal gonadotropin (hMG, Menopur^®^ 75 UI, Ferring^®^, Madrid, Spain) and synthetic serum substitute (SSS IrvineScientific^®^, Santa Ana, CA, USA). Oocytes were serially observed under the inverted microscope (Olympus, IX70, Tokio, Japan) 20, 24, 44, and 48 h after the IVM. Mature oocyte (MII) were considered to be those in which a rupture of the GV was observed, and the presence of a first polar corpuscle in the perivitelline space during the first 48 h of culture was observed under inverted optical microscope (Figure 5).

### 4.4. Oocyte Vitrification and Warming

We used vitrification/warming medium and the Cryotop^®^ open system device commercialized by Kitazato^®^ (BioPharma Co, Shizuoka, Japan). The vitrification/warming protocol used was the one recommended by the commercial company developed by Kuwayama [4]. This protocol was used in all the oocytes involved in this study, regardless of their maturation stage (GV or MII). Survival rate was evaluated microscopically with Hoffman contrast 2 h after warming and was based on morphology and integrity observations of the oocyte membrane.

### 4.5. Oocyte Fixation

For all groups (Group GV-Vit, Group MII-Vit, and Group not-Vit, see experimental design in Figure 1), the MII oocytes obtained by in vitro maturation [20–48 h] were fixed with 2% (*w/v*) paraformaldehyde solution (Electron Microscopy Sciences, Hatfield, PA USA), 0.5% (*w*/*v*) Tritón X-100 (Sigma-Aldrich^®^, Saint Louis, MO, USA), and 1μmol/L of Taxol^®^ (Paclitaxel, Sigma-Aldrich) in phoshate-buffered saline without calcium or magnesium, pH 7.4 (PBS, Biowest, Nuaillé, France) for 30 min at 37 °C and 5% (*v*/*v*) CO_2_. Oocytes where then washed three times for 15 min in PBS and stored in 2% (*w*/*v*) bovine serum albumin (BSA, Sigma-Aldrich), 0.1 mol/L of glycine (Sigma-Aldrich), 0.01% (*w*/*v*) Triton X-100, and PBS until they were immunolabeled (see Figure 1). All reagents were aliquoted in PBS except of Taxol, that as recommended by the commercial company was aliquoted in DMSO. 

### 4.6. Tubulin and Chromatin Staining

In order to observe the spindle conformation we performed the next methodology based on previous reports (7, 54, 79). All fixed oocytes were incubated with a mixture of two primary tubulin antibodies produced in mouse, anti-β-tubulin, and anti-α-tubulin (1:100) (Sigma-Aldrich) for 90 min. After that, the cells were washed three times for 10 min in PBS and incubated with the secondary antibody anti-mouse conjugated with Alexa Fluor 488 (1:500) (Jackson ImmmunoResearch, Ely, UK) for 1 h in the dark. Then, the oocytes were washed again three times for 10 min in PBS. For chromatin staining, we added 5μL of mounting medium with 4′,6-Diamidino-2-phenylindole dihydrochloride (DAPI, Vector Laboratories, Burlingame, CA, USA) and left for incubation for 5 min in the dark. All of the process was performed at room temperature. This part was performed at the Department of Biotechnology of the University of Alicante, Alicante, Spain.

### 4.7. Image Acquisition

A total of 199 oocytes were mounted using cavity slides, and the spindle characteristics and chromosome arrangement were evaluated by Confocal Laser Scanning Zeiss LSM 800 Microscope (Zeiss, Oberkochen, Germany) and Zeiss Imaging Software at the technical services of the University of Alicante. Z-stacks sections (1040 × 1040 pixels) of the entire spindle of each oocyte were obtained using an oil 40x objective and 408 nm and 561 nm lasers. Then, the sections were reconstructed using ZEN 2.5 lite software (Zeiss).

### 4.8. Statistical Analysis

The sample size was calculated to detect a minimum of 35% of difference in the proportion of oocytes with a disrupted plate between the control group (expected rate 15%) and any of the other experimental groups, with a confidence level of 95 (α = 5%) and a statistical power of 80% (β = 20%). The homogeneity of the groups was evaluated with the Kolmogorov–Smirnov test. The differences between the quantitative variables were verified using T-Test or Mann–Whitney U test. For the qualitative variables, the X^2^ test or Fisher’s test was used if both variables to be compared were dichotomous or some cell contained an expected frequency percentage of less than 5%. Comparison of Maturation Rates [MR = n° MII oocytes (24–48 h)/n° GV oocytes × 100], Survival Rate [SR = (n° 2 h viable devitrified oocytes/n° vitrified oocytes) × 100], and Normal Conformation Rate [NCR = n° oocytes with normal-partially normal conformation/n° oocytes evaluated) × 100], was performed using contingency tables, X^2^ test, with a level of α equal to 0.05, therefore, it was considered that there were significant differences when *p*-value was < 0.05.

## Figures and Tables

**Table 1:** Rates calculated according to the groups of study. Survival rate (SR), maturation rates (MR): MR by germinal vesicle breakdown (GVBD); after 24 h and 48 h. Average percentage by group and *p*-value. (*) *p*-value < 0.05 Group GV-Vit vs. Group MII-Vit. *(--) data not possible.*

		Group GV-Vit	Group MII-Vit	Group Not-Vit
SR		45/58 (77.6%)	30/44 (68.2%)	--
MR	GVBD	40/45 (88.9%) *	61/85 (71.8%) *	43/52 (82.7%)
	24 h	27/45 (60%)	56/85 (65.9%)	31/52 (59.6%)
	48 h	33/45 (73.3%)	65/85 (76.5%)	36/52 (69.2%)

**Table 2:** Normal spindle configuration and chromosomal position of oocytes depending on the in vitro maturation time (IVM), 24 or 48 h. * *p*-value < 0.05. IVM, in vitro maturation; CHR, chromosomes.

IVM	*n*	CHR	SPINDLE
24 h	61	24/61 (39.3%)	39/61 (63.9%) *
48 h	13	5/13 (38.5%)	12/13 (92.3%) *

## Data Availability

The data presented in this study are available in the article.

## Abbreviations

A	Abnormal
AR	Assisted reproduction
cAMP	Cyclic adenosine monophosphate
CHR	Chromosome
CRP	Cryoprotectants
DMSO	Dimethylsulfoxide
EG	Ethylene Glycol
EV	Embryo vitrification
GC	Granulosa cells
GV	Germinal vesicle (prophase I)
GVBD	Germinal vesicle breakdown
h	Hours
ICSI	Intracytoplasmic sperm microinjection
IVF	In vitro fertilization
IVM	In vitro maturation
MII	Metaphase II
MPF	Maturation promoting factor
MR	Maturation rate
N	Normal
OHSS	Ovarian hyperstimulation
OV	Oocyte vitrification
PA	Partially abnormal
PB	Polar body
PCOS	Polycystic Ovary Syndrome
SR	Survival rate
